# An Evolutionary View on Disulfide Bond Connectivities Prediction Using Phylogenetic Trees and a Simple Cysteine Mutation Model

**DOI:** 10.1371/journal.pone.0131792

**Published:** 2015-07-10

**Authors:** Daniele Raimondi, Gabriele Orlando, Wim F. Vranken

**Affiliations:** 1 Interuniversity Institute of Bioinformatics in Brussels, ULB-VUB, Brussels, Belgium; 2 Structural Biology Brussels, Vrije Universiteit Brussel, Brussels, Belgium; 3 Department of Structural Biology, VIB, Brussels, Belgium; 4 Machine Learning Group, ULB, Brussels, Belgium; Instituto de Biociencias—Universidade de São Paulo, BRAZIL

## Abstract

Disulfide bonds are crucial for many structural and functional aspects of proteins. They have a stabilizing role during folding, can regulate enzymatic activity and can trigger allosteric changes in the protein structure. Moreover, knowledge of the topology of the disulfide connectivity can be relevant in genomic annotation tasks and can provide long range constraints for *ab-initio* protein structure predictors. In this paper we describe PhyloCys, a novel unsupervised predictor of disulfide bond connectivity from known cysteine oxidation states. For each query protein, PhyloCys retrieves and aligns homologs with HHblits and builds a phylogenetic tree using ClustalW. A simplified model of cysteine co-evolution is then applied to the tree in order to hypothesize the presence of oxidized cysteines in the inner nodes of the tree, which represent ancestral protein sequences. The tree is then traversed from the leaves to the root and the putative disulfide connectivity is inferred by observing repeated patterns of tandem mutations between a sequence and its ancestors. A final correction is applied using the Edmonds-Gabow maximum weight perfect matching algorithm. The evolutionary approach applied in PhyloCys results in disulfide bond predictions equivalent to Sephiroth, another approach that takes whole sequence information into account, and is 26–29% better than state of the art methods based on cysteine covariance patterns in multiple sequence alignments, while requiring one order of magnitude fewer homologous sequences (10^3^ instead of 10^4^), thus extending its range of applicability. The software described in this article and the datasets used are available at http://ibsquare.be/phylocys.

## Introduction

Disulfide bonds are covalent links between the thiol groups of cysteine residues. In proteins, the formation of correct disulfide bonds is very relevant during the folding process, as they pose conformational constraints that destabilize the unfolded state and create favourable enthalpic interactions in the native state [1]. In addition, disulfide bonds can regulate enzymatic activity by explicitly performing catalytic duties or triggering allosteric changes in the structure [2]. However, a large number of known protein sequences lack experimentally determined 3D structures, and no disulfide bond information is available. In these cases prediction of the disulfide connectivity pattern helps to structurally characterize the sequence [2, 3]. In particular, disulfide bond patterns can help to recognise proteins with similar folds independently of their sequence similarity [4, 5], and in *ab-initio* structure predictors they provide valuable long-distance constraints that reduce the overwhelming conformational space these algorithms have to search [4, 6].

The existing bioinformatics methods that predict cysteine behaviour and connectivity from protein sequence typically work in two stages: in the first step they predict the cysteine oxidation states, followed by a second step where the disulfide connectivity patterns are inferred [7–11]. The prediction of the cysteine oxidation states and the connectivity patterns are two very different problems. More satisfactory performances are obtained for the former, with 93% of cysteine oxidation states correctly classified and globally 86% of proteins correctly predicted [10] using Machine Learning (ML) methods ranging from Support Vector Machines (SVM) to Recurrent Neural Networks (RNN) or Graphical Models such as Conditional Random Fields (CRF) and their derivatives [7–12]. The latter problem is more challenging, with on average only around 50% of proteins predicted with the correct disulfide connectivity. Commonly ML methods are applied in this step [7–11, 13, 14], sometimes in combination with *unsupervised* predictors [9, 15, 16].

These unsupervised predictors can provide information that is in part orthogonal to the model learned by ML methods [15, 16] and can improve the performances of such supervised methods by up to ∼10% [9, 16]. Typically, unsupervised predictors are based on statistical methods such as Mutual Information or Direct Coupling Analysis (DCA) [9, 17, 18] that infer disulfide connectivity patterns from the covariance between cysteine-harboring positions in Multiple Sequence Alignments (MSA) of collected homologs. The underlying assumption, widely adopted in the field of Contact Prediction (CP), is that residues spatially close in the native structure tend to co-evolve during evolution in order to preserve their interaction [17, 19], resulting in compensatory mutations. Methods derived from the CP literature have indeed been successfully applied to disulfide connectivity prediction; two of the most recent unsupervised methods are ICOV and MIp [9]. These approaches are effective but do not take into account the evolutionary distances between the sequences, a problem we previously addressed in Sephiroth [16], an unsupervised predictor that weighs the effect of tandem compensatory mutations in function of the evolutionary distance between the proteins in which they occur, so providing a significant performance improvement.

In this work we describe PhyloCys, which derives the evolutionary history of the target protein based on its complete sequence, and uses the presence of cysteines in the proteins’ ancestral sequences to predict disulfide connectivity patterns. PhyloCys so avoids looking only at the compensatory mutations observed between sequences at the same “evolutionary level” (the homologs in the MSAs), and instead employs the information in a phylogenetic tree reconstructed with ClustalW [20] from MSAs generated by HHblits [21]. By assuming that cysteine mutations are more likely to be lost, not gained, in evolution, the cysteine positions in ancestral sequences in the tree are inferred and subsequently interpreted by a simplified model of cysteine evolution. The performance of PhyloCys in predicting disulfide bond connectivity is similar to Sephiroth [16] and improves over CP-based methods by 26–29%, while requiring one order of magnitude fewer homologous sequences (10^3^ instead of 10^4^).

## Methods

### Datasets

#### PDBCYS and SPX datasets

We based the development of our tool on two widely used datasets that contain disulfide bond information for proteins: PDBCYS [10] and SPX [11]. PDBCYS was used during the development of ICOV and MIp [9] and thus permitted us to directly compare to the prediction performances of these methods. This dataset contains 1797 protein sequences obtained from the PDB release of May 2010. 276 proteins in it contain only oxidized cysteines, 1320 proteins contain only cysteines in reduced form and 201 sequences contain cysteines in both states. PDBCYS has 100 proteins with 2 bonds, 85 proteins with 3, 41 with 4 and 37 with 5. The SPX dataset contains 1018 sequences; of which 398 have 2 bonds, 211 have 3 bonds, 219 have 4 bonds and 88 have 5 bonds (see S2 Fig. for a comparison of the distributions of disulfide bonds).

The PDBCYS and SPX datasets have 70 proteins in common: in order to use SPX as independent validation set for testing the validity of the biological assumptions postulated by our method, we removed these 70 shared sequences, obtaining a subset of 948 proteins called IND-SPX. A comparison between PDBCYS and SPX sequence lengths is shown in S3 and S1 Figs. The differences between SPX and PDBCYS are extensively discussed in the Results section.

#### New OXCYS15 and OXCYSnr datasets

To further validate to our method and to provide the community with an up-to-date benchmark, we designed two new datasets with disulfide bond information based on the April 2015 version of the PDB. The OXCYS15 dataset (http://dx.doi.org/10.6084/m9.figshare.1422073) contains all the PDBs deposited after May 2010 and is temporally independent from PDBCYS, which was built in May 2010 [10]. The OXCYSnr dataset (http://dx.doi.org/10.6084/m9.figshare.1422074) is on the other hand evolutionarily independent and contains only proteins with no homologs in the PDBCYS and SPX datasets. We produced these datasets by first extracting all entries from the PDB containing proteins with at least two cysteines. The disulfide bonds annotations for these sequences were taken from the header of the PDB file and mapped to the sequence using the SIFTS resource [22]. We then filtered these sequences by removing proteins shorter than 40 residues or longer than 1500, by removing entries released before June 2010 (OXCYS15), and by removing proteins homologous to SPX or PDBCYS based on the absence of results for BLAST searches with an E-value cutoff of 10^−7^ (OXCYSnr). Finally, we independently clustered the remaining sequences in both OXCYS15 and OXCYSnr with BLASTCLUST in order to reduce the internal homology in both databases. The parameters used were 20% of maximum sequence identity at 90% of coverage and, for each cluster obtained, we took only the first sequence. We annotated as oxidized only the cysteines involved in intra-chain disulfide bonds and we labeled as not-oxidized the ones involved in inter-chain disulfide bonds.

**Fig 1:**
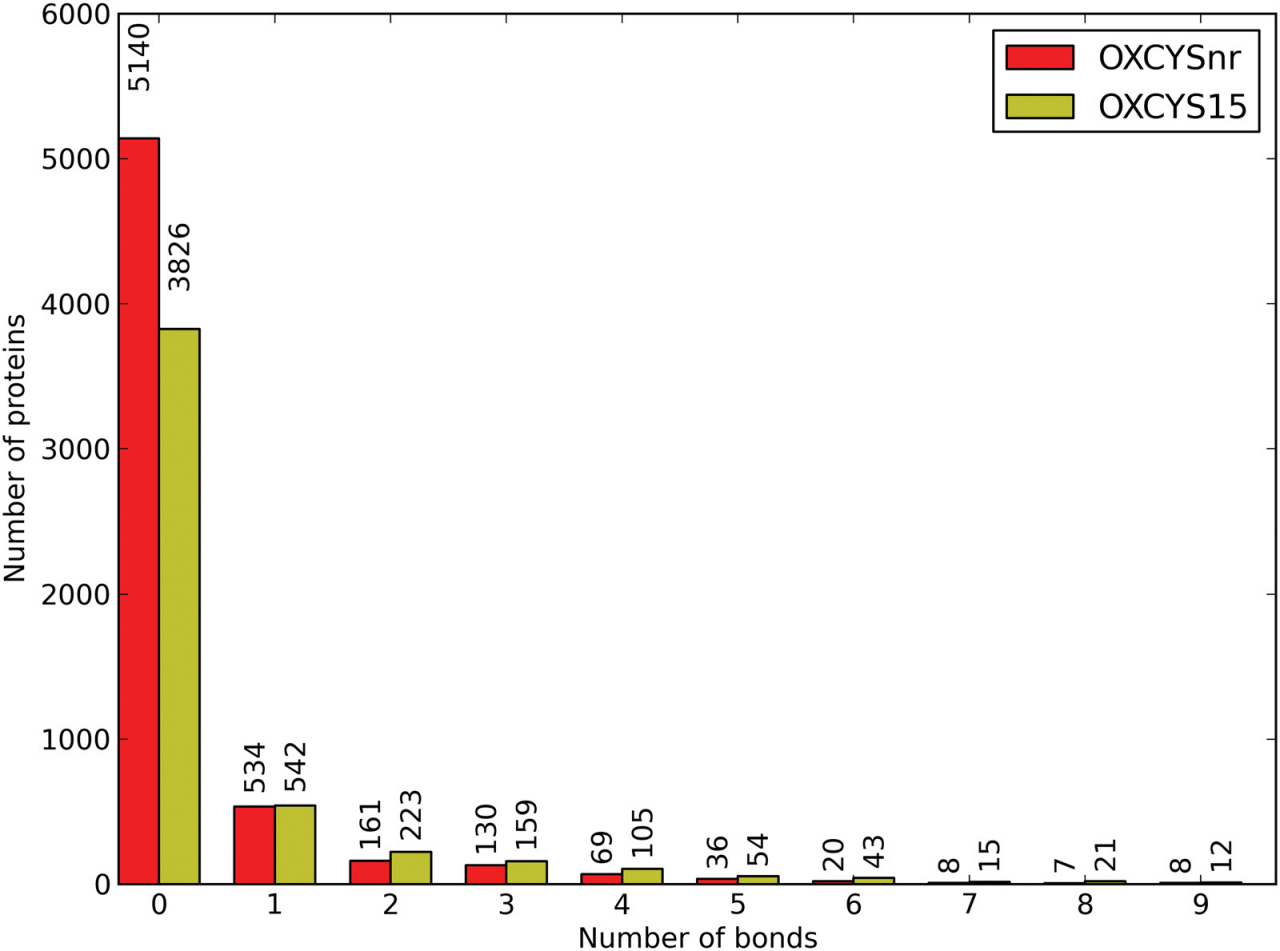
Distribution of the bonds in OXCYSnr and OXCYS15 datasets.

After these steps, OXCYSnr contains 6118 proteins, with 21994 reduced and 4136 oxydized cysteines. The majority of the sequences, 5140, have only reduced cysteines. The remaining are divided as follows: 534 proteins have 1 disulfide bond, 161 proteins have 2 bonds, 130 proteins have 3 bonds, 69 proteins have 4 bonds and 36 proteins have 5 bonds. The maximum number of bonds in a protein is 15 (Data shown in red in Fig 1). OXCYS15 contains 5020 proteins, with 19728 reduced cysteines and 6118 oxidized ones. Again, most of the proteins (3826) have only cysteines in reduced form, while 542 proteins have 1 bond, 223 proteins have 2 bonds, 159 proteins have 3 bonds, 105 proteins have 5 bonds and 54 proteins have 5 bonds. The proteins with the most complex disulfide connectivity has 24 bonds (Data shown in yellow in Fig 1). Both datasets are publicly available from Figshare, at http://ibsquare.be/phylocys and from the git repository.

### PhyloCys

The PhyloCys algorithm starts from a sequence (called the “target” or “query” sequence) in which some cysteines are known to be involved in disulfide bonds. We here describe the procedure to predict the connectivity of a single sequence, where *C* = (*c*_1_, *c*_2_, …, *c*_*n*_) is the list of oxidized residue positions in the query sequence and *d* = ∣*C*∣/2 is the number of disulfide bonds in the protein.

#### Homologs retrieval and tree building

In the first step homologous sequences are collected with HHblits [21] using its standard database, a processed version of Uniref20. The obtained MSA, which represents the protein family of the query sequence, is reformatted and filtered so that only the homologous sequences are retained that have an even number of cysteines present in the oxidized positions *C* = (*c*_1_, *c*_2_, …, *c*_*n*_) of the query sequence. This filtering involves only the collected homologs and does not have any restrictions about the total number of cysteines in the query protein or the homologs. This filtering removes noise due to spurious single mutations which disrupt the tandem-mutation signal. In the next step, ClustalW [20] is applied to the filtered MSA to obtain a neighbor-joining distance-based phylogenetic tree in Newick format. In our Python implementation, the Newick-formatted trees are read and processed using the ETE2 library [23]. Finally, we root the phylogenetic trees by explicitly adding a sequence that is very distant from the others; this ‘outgroup sequence’ branches outside of the tree of interest and provides a consistently defined root for the phylogenetic tree. We created the outgroup sequence by randomly shuffling the query sequence and adding it as last sequence of the MSA before building the tree with ClustalW.

#### Labeling and traversing the internal nodes

In a phylogenetic tree, the leaves correspond to the input MSA sequences, which currently exist, whereas the inner nodes represent ancestral sequences. The simplified evolutionary model in PhyloCys works off the cysteine conservation state, and infers which oxidized cysteines are conserved in each inner node. First, each sequence from the input MSA (leaves) is given a binary non-unique label relating to the presence of cysteines: each position represents the presence (1) or absence (0) of a cysteine residue in the oxidized positions *C* = (*c*_1_, *c*_2_, …, *c*_*n*_) as observed in the query sequence. Starting from each leaf, our simplified model of evolution climbs up the tree until the root is reached, and infers the binary label of each inner node. The implemented recursive algorithm works as follows: starting from a leaf *L*_*i*_ in a binary phylogenetic tree, the algorithm visits *L*_*i*_’s parent, *F*_*i*_ = *up*(*L*_*i*_), and assigns to it a binary label that is built by taking the union of the 1s in *F*_*i*_’s children, *L*_*i*_ and its *siblings* *L*_*k*_.

**Fig 2:**
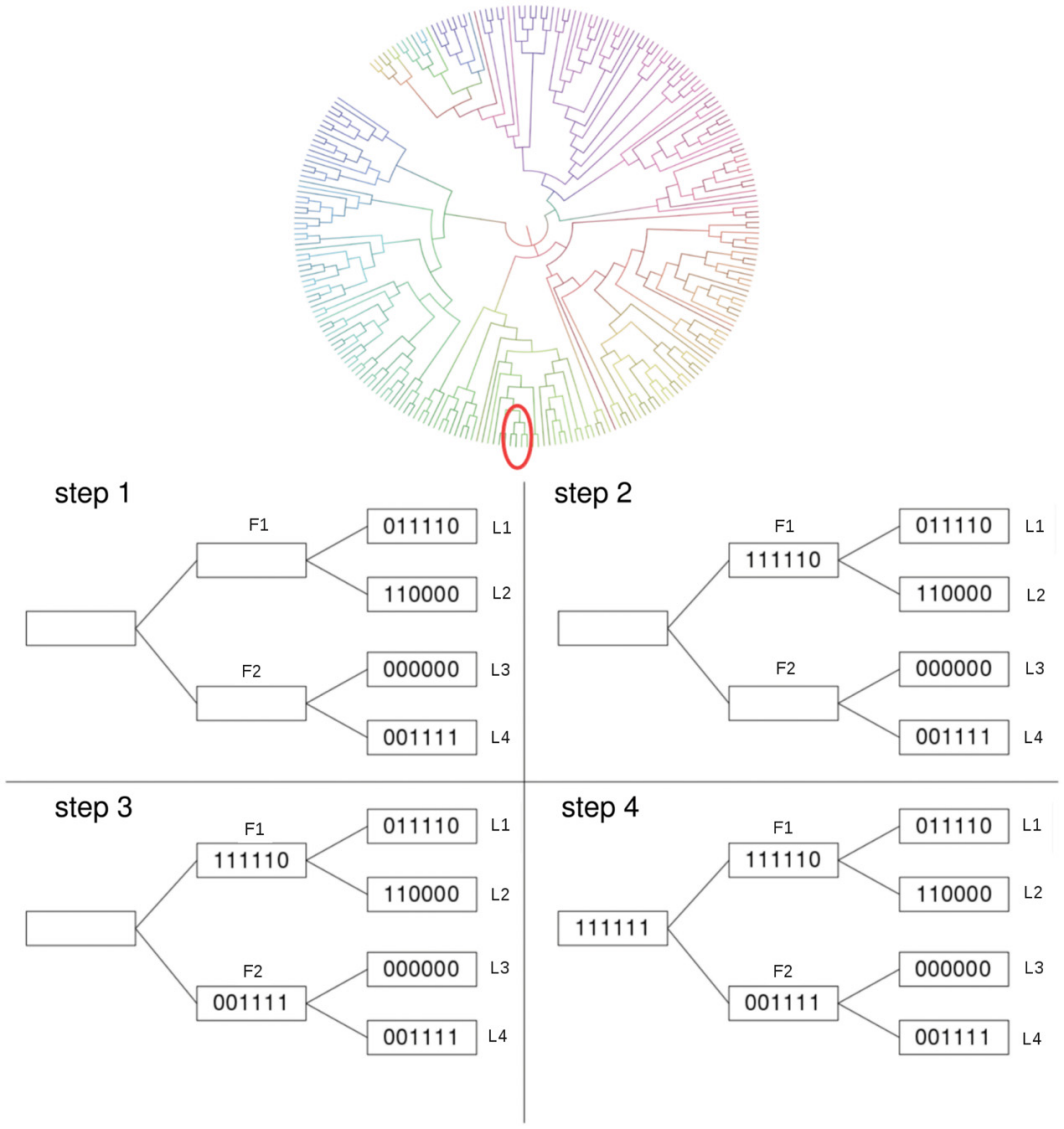
Illustrative example of the naming procedure. An example of the procedure used by PhyloCys to assign binary names to the inner nodes of a small sub-tree (the red circle) of the cladogram representing the phylogenetic tree is shown in four steps. In the initial state of the tree (1), only leaves (the observed homologs) have labels assigned to them. Starting from the topmost leaf *L*_1_, the tree is traversed until the root is reached. At step 2 the label of *L*_1_’s parent *F*_1_ is assigned, by taking the OR between *L*_1_ and *L*_2_ names. In step 3 the label to *F*_1_’s father (the root of the subtree) has to be assigned. Since the other child of the root has no label assigned to it, the recursive procedure visits that sub-tree and returns the correct binary assignment. In the last step 4, the union of the ones in *F*_1_ and *F*_2_ is computed and assigned to the root of this sub-tree.

The algorithm then visits *F*_*i*_’s parent, *G*_*j*_ = *up*(*F*_*i*_) and assigns a label to it in the same way as before, by computing the binary operator OR between *G*_*j*_’s children, *F*_*i*_ and *F*_*j*_. If *F*_*j*_ has no binary label assigned to it, the algorithm recursively assigns labels to the *F*_*j*_ branch of the tree by going down it until the leaves are reached and then backtracking. This procedure stops when the root is reached and is repeated for each leaf in the tree. An illustrative example of this procedure is shown in Fig 2; note that the root always has all oxidised cysteines present. This corresponds to a *maximum parsimony* approach since it implies the smallest possible number of mutations in each step.

The two assumptions underlying this model are that (1) the disulfide bond connectivity pattern is conserved within the protein family (the pool of closely related proteins in the HHblits MSA), as structure tends to be more conserved than sequence and related homologs are likely to share many structural features, and (2) during the evolution of our *sampled* protein family, cysteines in the oxidized state can only be lost. If the mutation of a bonded cysteine occurs with probability *p*, another mutation in the same position that restores a previously existing cysteine must occur with a probability *p* × *u* ≪ *p*, where *u* is the probability of mutating back the position to restore the cysteine. This is in line with the tandem mutation model, where evolutionary pressure caused by the mutation of an oxidized cysteine makes mutations of the coupled cysteine more likely, whereas restoration of a fully lost disulfide bond requires two mutations to cysteines. This is a rare event, unlikely to occur within a protein family in our limited model. We discuss some examples of validation of these assumptions in the Results section.

#### Inferring the connectivity

After the assignment of the binary cysteine labels to every node in the tree, the algorithm again visits the tree and infers the pattern of co-variation among oxidized cysteine positions. For each leaf it climbs up the tree until the root is reached, storing the binary labels of each node in an ordered list (*L*_*i*_, *F*_*i*_, *G*_*i*_, …, *ROOT*). A binary label *l*_0_ with 0s in every position is added to the list before the first position, that is the leaf from which the upward visit of the tree started.

**Fig 3:**
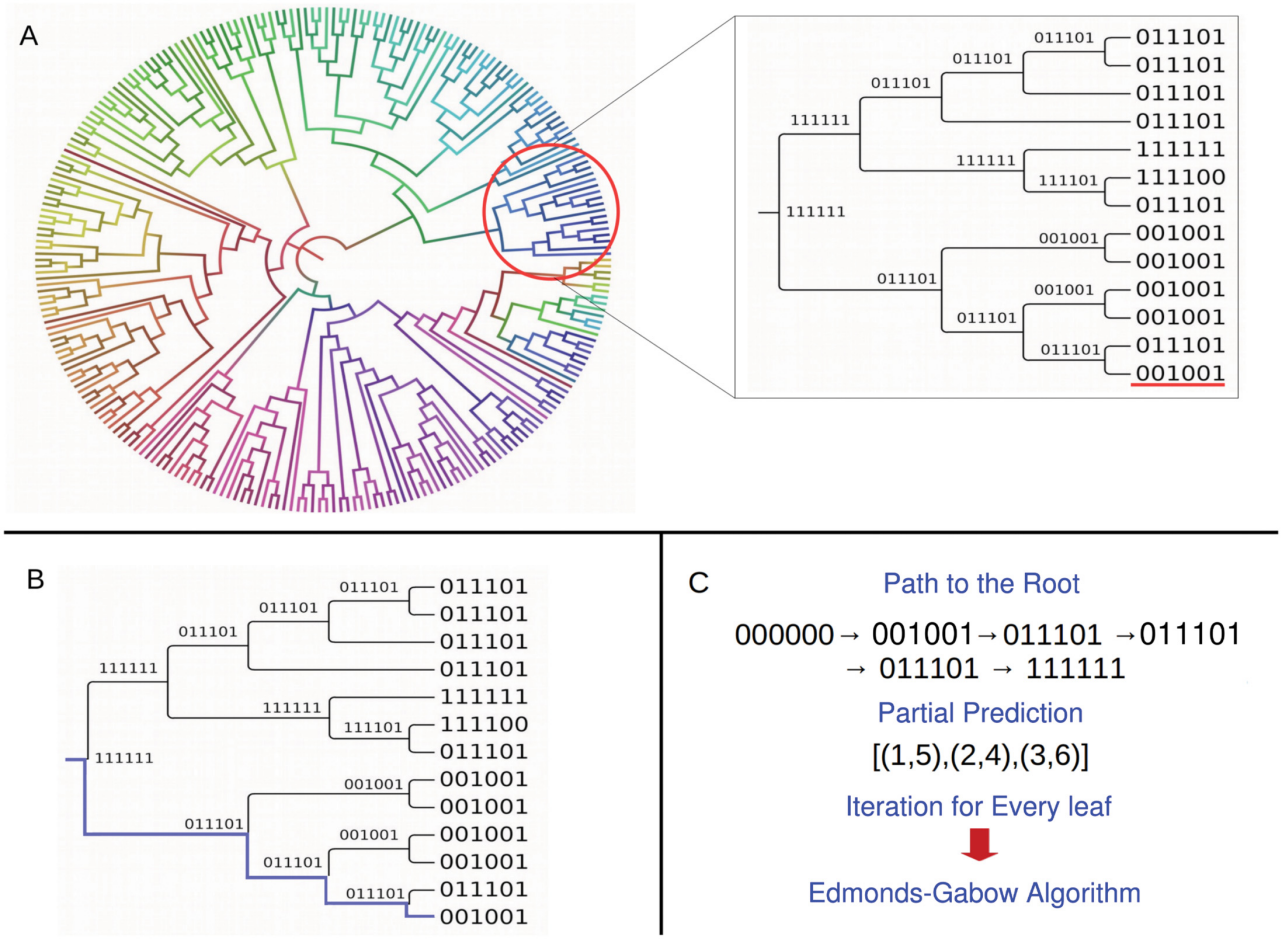
Illustrative example of the PhyloCys algorithm for inferring disulfide bonds from patterns of co-variation among lineages. Given a phylogenetic tree and the binary labels assigned to each inner node (A), the path from the last leaf to the root is highlighted in (B). The sequence of nodes encountered from the leaf to the root (C) is used to assign weights to the connectivity graph and the most likely connectivity pattern is obtained with Edmonds-Gabow algorithm (C).

This new list Λ = (*l*_0_, *l*_1_, *l*_2_, …, *l*_*root*_) is then analyzed by considering the changes in the patterns of ones and zeroes occurring between any two adjacent elements *l*_*i*_ and *l*_*i*+1_ (a node and its parent). An illustrative example of this procedure is shown in Fig 3.

Every change observed between the binary labels is indicative of the cysteines’ degree of co-mutation during the evolutionary history of the protein family. These changes are analyzed in order to assign weights to an undirected and fully connected *connectivity graph* 𝔾 = (*C*, *β*), which represents all the possible connectivity patterns between the ∣*C*∣ oxidized cysteines, here represented as nodes. The possible edges are ∣*β*∣ = ((∣*C*∣ × (∣*C*∣ − 1))/2), representing all disulfide bond possibilities.

The weights in the connectivity graph 𝔾 are assigned based on comparison of all binary label pairs *l*_*i*_ and *l*_*i*+1_. For each such pair, ℙ(*l*) describes the parity of the binary label *l*, which is 1 if *l* contains an even number of ones (even parity) and odd if the number of 1s is odd (odd parity). We differentiate between 2 main cases listed below:

- *l*_*i*_ = *l*_*i*+1_: the two binary labels are identical; no changes occurred between these two nodes and thus no inferences can be made. The algorithm increases *i* by one and goes to the next step.
- ℙ(*l*_*i*_) = ℙ(*l*_*i*+1_): if the parity of the labels is the same, but the labels are not identical, then one or more pairs of cysteines experienced a tandem mutation in the step between *l*_*i*_ and *l*_*i*+1_. Positive weights are in this case added to all the edges of 𝔾 that connect the cysteines involved in these changes. If only a single pair of positions of *l*_*i*_ mutated, for example *p*_2_ and *p*_4_, the corresponding edge (in this example (2, 4) ∈ *β*) will be incremented by one. If more than two positions of *l*_*i*_ are mutated, for example *p*_1_, *p*_2_, *p*_3_ and *p*_4_, then it is not possible to know the precise pairing of the disulfide bonds, in essence because the *time resolution* of the *evolutionary sampling* in the phylogenetic tree is not high enough to separate the related mutation events. In this case we assign a positive weight to each possible disulfide bond between the *μ* mutated cysteines by increasing each of the possible bonds (totalling (*μ* × (*μ* − 1))/2) by 1/((*μ* × (*μ* − 1))/2) = 2/(*μ* × (*μ* − 1)).

When the root of the tree is reached, the procedure is repeated for the next leaf until all the leaves have been processed. At this point the edges of 𝔾 have been updated with all the information available.

After the inference of the weights we followed the approach proposed in [14], applying the Edmonds-Gabow algorithm [24] to 𝔾 for maximum weight perfect matching and so obtain the most likely disulfide connectivity pattern.

The algorithm described so far is publicly available at: http://ibsquare.be/sephiroth.

### Performance Evaluation

We use the most commonly used indexes in the disulfide connectivity prediction literature, *R*_*b*_ and *Q*_*p*_, to evaluate our method. They are defined as:

- $R_{b}=\frac{B_{p}}{B_{o}}$ is the number of correctly predicted disulfide bonds *B*_*p*_ divided by the total number of observed bonds *B*_*o*_ among the predicted proteins.
- $Q_{p}=\frac{P_{p}}{P_{o}}$ is the number of proteins with a completely correctly predicted connectivity *P*_*p*_ divided by the total number of proteins *P*_*o*_ for which the connectivity prediction was attempted.

### Benchmark with Machine Learning predictor

We added the unsupervised PhyloCys predictions to a supervised predictor reproduced from literature to assess whether our method can contribute to the performance of Machine Learning-based methods. We implemented the approach adopted in [9–11] with the sklearn library [25] using a Support Vector Regressor (SVR) learner with RBF kernel with the default library parameters *C* = 1.0 and *γ* = 0.5. The standard feature vectors have 523 dimensions (as in [9, 10]) that encode every possible pair of oxidized cysteine residues:

- For a sequence window of 13 positions long, 6 left and 6 right with respect to the central oxidized cysteine, each pair of cysteines is represented by 20 × 2 × 13 = 520 dimensions. The variability of each position within this window is encoded by 20 dimensions corresponding to each natural amino acid and representing the column of the MSA extracted from HHblits alignments obtained with 3 iterations and E-value = 10^−2^.
- The cysteine sequence separation is encoded in 1 dimension. For each pair of cysteines at positions (*c*_*i*_, *c*_*j*_), their separation is given by log(∣*c*_*i*_ − *c*_*j*_∣).
- The relative order of the cysteines in the pair is encoded in 2 dimensions. Given *n* cysteines *C* = (*c*_1_, *c*_2_, …, *c*_*n*_) in the query protein, their relative order *O* is calculated as *O* = (*c*_1_/*n*, *c*_2_/*n*, …, *c*_*n*_/*n*).

For each protein with ∣*C*∣ oxidized cysteines at positions *C* = (*c*_1_, *c*_2_, …, *c*_*n*_), all the (∣*C*∣ × (∣*C*∣ − 1))/2 feature vectors representing the possible disulfide bonds were built and provided to the SVR algorithm. The real values obtained as output predictions are used as weights in a connectivity graph and the most likely connectivity pattern is calculated with Edmonds-Gabow (EG) algorithm. For each feature vector representing a pair of oxidized cysteines (*c*_*i*_, *c*_*j*_), PhyloCys predictions are added as a single dimension binary value: 1 if (*c*_*i*_, *c*_*j*_) is predicted to be bonded and 0 otherwise. These results are shown in Table 5 and discussed in the Results section; the skSVR+phyloCys and skSVR+sephiroth feature vectors have 524 dimensions, the skSVR+phyloCys+sephiroth ones have 525 dimensions.

**Table 5:** Performances of unsupervised disulfide connectivity predictors when added to supervised Machine Learning methods.

Method	Number of Bonds								
	2		3		4		5		Average
	Rb	Qp	Rb	Qp	Rb	Qp	Rb	Qp	Qp
Random	33	33	20	7	14	1	11	0.1	15
SVR	75	75	60	48	57	44	46	19	54
SVR+MIp+ICOV	76	76	63	55	68	51	59	32	59
skSVR	79	79	67	60	60	41	55	28	60
skSVR+Sephiroth	86	86	71	64	67	50	66	46	68
skSVR+PhyloCys	82	82	68	59	69	59	68	51	67
skSVR+Sephiroth+PhyloCys	87	87	69	61	74	62	70	49	69

Performances of different supervised Machine Learning-based methods on the PDBCYS dataset. SVR and SVR+MIp+ICOV scores are reported from [9]; Sephiroth performances have been obtained with 3 iter E-value = 10^−2^ HHblits MSAs and are reported from [16], PhyloCys scores are obtained with 3 iter E-value = 10^−5^.

## Results

### Performances in function of the Multiple Sequence Alignments

The predictive ability of PhyloCys is dependent on the quality and extent of the phylogenetic tree, which is in turn based on the availability of sequences from the same protein family as identified by remote homology search methods such as HHblits. In order to show the degree of dependence of our method on the parameters used to build the MSAs, we varied the HHblits “number of iterations” and “E-value threshold” values. Table 1 shows that the trend of the Qp scores averaged over the proteins with 2–5 bonds is reasonably stable but tends to increase with more iterations. Even with the highest number of iterations, for some sequences HHblits provides empty MSAs and no phylogenetic tree can be constructed. The connectivity is in these cases guessed randomly in order to provide a fair performance comparison. The best score is obtained with the MSAs obtained with 4 iterations and E-value = 10^−5^. From Table 1 it is also clear that PhyloCys performs much better than a random predictor, even for the sub-optimal HHblits parameters.

**Table 1:** PhyloCys performances in function of the MSAs.

Alignments		Number of Bonds									MSAs Average Size	Empty MSAs
		2		3		4		5		Average		
Num. Iter.	E-value	Rb	Qp	Rb	Qp	Rb	Qp	Rb	Qp	Qp		
Random		33	33	20	7	14	1	11	0.1	15		
1	10^+1^	74	74	52	42	56	39	35	14	50	1035	2
1	10^−1^	71	71	54	44	61	42	36	11	49	963	2
2	10^−5^	72	72	55	46	57	42	42	30	53	1070	3
3	10^−2^	68	68	58	51	56	37	45	24	51	1406	3
3	10^−5^	73	73	55	45	64	51	40	27	**54**	1195	3
4	10^−5^	73	73	56	46	57	44	40	24	53	1256	3

Variation of the PhyloCys performance on the PDBCYS dataset in function of different alignment parameters (number of iterations and E-value cut-off).

**Fig 4:**
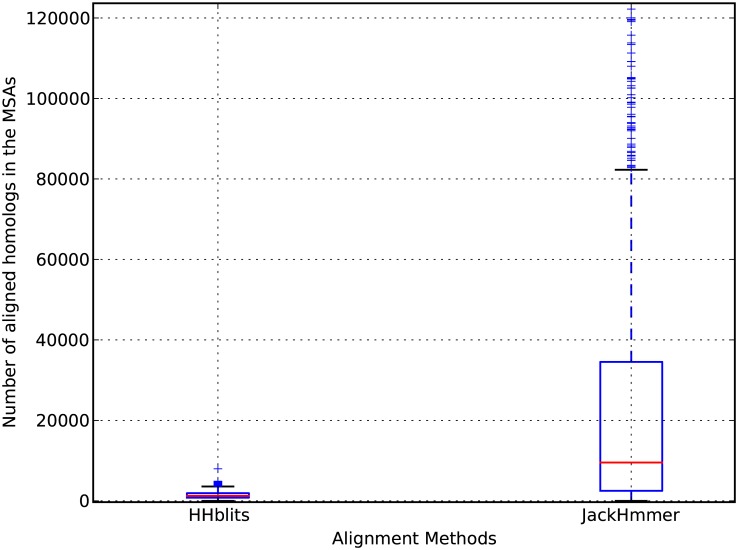
Comparison between the distribution of the number of homologs retrieved with HHblits and JackHmmer. This boxplot shows the distributions of the number of homologs optimal for PhyloCys (HHblits alignments, on the left) versus the ones required by MIp and ICOV (JackHmmer alignments, on the right).

The HHblits-generated MSAs used by PhyloCys have on average around 1000 sequences each (rightmost column in Table 1). We compared the PhyloCys alignments to the JackHmmr based alignments [26] (with 3 iterations on NCBInr). Fig 4 shows the comparison between the number of homologs retrieved by both approaches, and illustrates that there is a large difference between the sizes of the MSAs produced by them: the furthest outlier of the HHblits distribution has less homologs than the median of the MSAs obtained with JackHmmer. We further assessed whether these alignments have significantly different sizes using a Wilcoxon signed-rank test and a Student’s t-test, obtaining p-values of respectively 1.22 × 10^−241^ and 1.8 × 10^−97^. This shows that the number of homologs used by PhyloCys is significantly lower than other state of the art methods, by about an order of magnitude. The MSAs are therefore much less demanding in terms of required homologous sequences, and this is favourable for the computationally expensive step of building phylogenetic trees, in case of PhyloCys with ClustalW [20]. In particular the non-heuristic neighbor-joining approach appears to be Θ(*n* ^3^) [27] and in general these algorithms lead to at least NP-complete problems [28], making their application on jackHmmer MSAs computationally challenging. The number of homologs is important in determining the performance of the method, however: S4 Fig. shows the trend of the Qp performances for 2 to 5 bonds plus their weighted average in function of subsets of the total available homologs for the PDBCYS dataset based on HHBlits with 3 iterations and E-value = 10^−5^. While the Qp scores for the single bonds show some variability, the Average Qp shows a relatively steady 26% increase from 10% to 100% of homologs included.

### Comparison with other methods

The PDBCYS dataset allows a straightforward comparison between the performance of PhyloCys and the state of the art unsupervised disulfide connectivity predictors MIp and ICOV [9]. Since all these predictors are unsupervised, no learning is performed and cross-validation is in principle not required. However, we did reproduce the averaged cross-validation over 20 subsets used in ICOV [9], where it provides results coherent with supervised methods in the rest of the paper (Table 2). PhyloCys with the best performing alignment (3 iterations with E-value = 10^−5^) provides a +27.3% improvement with respect to ICOV and +24.4% with respect to MIp, with performances comparable to the Sephiroth method we previously developed [16] (Table 2). Compared to the best MIp or ICOV scores in function of the number of bonds, PhyloCys provides a +15% improvement for 2 bonds, +5% for 3 bonds, +69% for 4 bonds and +81% for 5 bonds. The PhyloCys prediction improvements therefore increase with rising number of cysteine bonds; these are the most difficult cases to predict, as is clear from the sharply decreasing random prediction scores [14]. With respect to Sephiroth, PhyloCys performs on average 1.8% less, but analyzing the scores in function of the number of bonds, we outperform it in proteins with 2, 4 and 5 bonds.

**Table 2:** PhyloCys performances compared to other unsupervised predictors.

Method	Number of Bonds								
	2		3		4		5		Average
	Rb	Qp	Rb	Qp	Rb	Qp	Rb	Qp	Qp
Random	33	33	20	7	14	1	11	0.1	15
ICOV	62	62	53	42	52	27	40	16	44
MIp	68	68	48	38	49	29	34	14	45
Sephiroth^a^	77	77	61	53	63	45	44	24	57
PhyloCys^a,b^	78	78	54	45	59	44	40	27	55
PhyloCys^a,c^	78	78	53	44	63	49	40	29	56

Performances of different unsupervised methods on the PDBCYS dataset. MIp and ICOV scores are reported from [9] and Sephiroth scores from [16].

^*a*^ The predictions were averaged over the 20 fold division provided with the dataset.

^*b*^ HHBlits 4 iter E-value = 10^−5^.

^*c*^ HHBlits 3 iter E-value = 10^−5^.

The 20-fold cross-validation-like procedure has the main drawback that the scores averaged in this way can be influenced by sampling imbalances, especially because the majority of the folds contain few proteins for each number of bonds. For example, each fold contains on average 5 proteins with 2 bonds, 4 with 3 bonds, 2 with 3 bonds and 2 with 5 bonds, so if some proteins in a fold are predicted fully correct or completely wrong, it is relatively easy to obtain extreme (100% or 0%) Qp scores. When comparing PhyloCys performances obtained by averaging the scores over the entire dataset (Table 1) with the one obtained with the 20 fold averaging (Table 2) this sampling bias is responsible for a +3.7% apparent improvement of performances.

### Validation on the SPX dataset

In order to further assess PhyloCys, both with regard to its capability to generalise over different sets of proteins and to the validity of the model, we also validated on the IND-SPX dataset (Table 3), which is the SPX dataset [11] where the 70 sequences that are also present in PDBCYS were removed. The Qp scores are in general lower than the scores obtained on PDBCYS, but in all cases they remain markedly higher than random prediction. The most obvious reason for this decrease in performance is the distribution of protein lengths in IND-SPX compared to PDBCYS (S3 and S1 Figs): the SPX dataset contains more shorter proteins than PDBCYS, with an average sequence length of 197 compared to 247 in PDBCYS. Moreover, the shortest protein is 13 residues long in SPX, and 40 residues in PDBCYS.

**Table 3:** PhyloCys performances on the IND-SPX dataset.

	Dataset		Number of Bonds								
			2		3		4		5		Average
Methods	Dataset	MSAs	Rb	Qp	Rb	Qp	Rb	Qp	Rb	Qp	Qp
Random			33	33	20	7	14	1	11	0.1	15
PhyloCys	IND-SPX	3 iter 10^−5^	60	60	34	26	37	18	44	18	37
PhyloCys	IND-SPX	4 iter 10^−5^	59	59	33	24	39	18	46	23	36
Sephiroth	IND-SPX	3 iter 10^−2^	60	60	38	30	35	21	47	23	39
Sephiroth	IND-SPX	3 iter 10^−5^	58	58	32	25	36	22	40	18	36
PhyloCys	IND-SPXnoFrag	3 iter 10^−5^	75	75	51	40	39	20	44	18	47
PhyloCys	IND-SPXnoFrag	4 iter 10^−5^	73	73	50	37	41	20	46	23	46
Sephiroth	IND-SPXnoFrag	3 iter 10^−2^	78	78	54	45	38	23	47	23	51
Sephiroth	IND-SPXnoFrag	3 iter 10^−5^	76	76	50	40	40	24	40	18	49

Comparison of PhyloCys performances on the IND-SPX dataset and the IND-SPXnoFrag datasets with Sephiroth performances, reported from [16].

**Fig 5:**
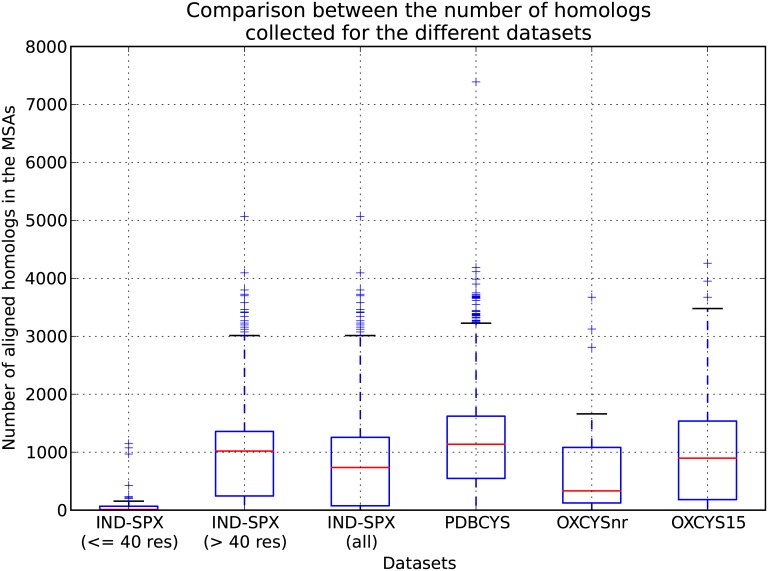
Distribution of the number of collected homologs among the datasets. Boxplot showing the distributions of the number of homologs retrieved by HHblits among datasets and their subsets. For the sequences shorter than 40 residues in IND-SPX too few homologs are collected to yield reliable predictions. The IND-SPX distribution has in general fewer collected homologs with respect to PDBCYS, but removing the sequences shorter than 40 residues from IND-SPX shifts the resulting IND-SPXnoFrag distribution towards higher median values. This also leads to a 25% improvement on the prediction performances (Table 3). The OXCYS15 dataset has a median number of homologs similar to PDBCYS and IND-SPXnoFrag, but has slightly higher variance. OXCYSnr has the smallest number of homologs available with the smaller variance (except for the fragments), resulting in lower performances on this dataset (Table 4).

This difference in sequence length is reflected in the distributions of the MSA sizes produced by HHblits for the sequences in, respectively, PDBCYS, IND-SPX, in the subset of IND-SPX with sequences shorter than 40 residues (IND-SPXfrag) and in its subset with sequences longer this threshold (IND-SPXnoFrag) (Fig 5). For only a few outlier proteins in IND-SPXfrag a number of homologs comparable with the median of the other datasets were retrieved; the entire distributions lies below 100 homologs and the median is around 20 (S5 Fig). The IND-SPXnoFrag set on the other hand results in an MSA size distribution closer to the one obtained from PDBCYS. In Table 3 it is possible to notice that this upwards shift in the number of homologs is responsible for a +27% improvement of the predictive performances for both selected configurations of alignment parameters. From this analysis it thus appears that the decrease of the performances on the SPX dataset is mainly due to the inclusion of many short proteins, which leads to a sub-optimal collection of homologs by HHblits and subsequently a poor build of the phylogenetic trees. Table 3 also shows the comparison of PhyloCys with Sephiroth on both IND-SPX and IND-SPXnoFrag. Both methods perform similarly on the more difficult IND-SPX dataset, resulting in a roughly 30% performance drop compared to PDBCYS (see Table 2), while on the IND-SPXnoFrag dataset Sephiroth performs 8% better than PhyloCys with its best parameters and 4% better using the same MSAs.

### Validation on the OXCYSnr and OXCYS15 datasets

We provided further validation to our method by building two new datasets from the April 2015 version of PDB (see Methods). OXCYS15 contains proteins whose experimental structure has been deposited at the PDB after the creation of PDBCYS (June 2010), while OXCYSnr contains only proteins that share no homology with entries of both PDBCYS and SPX. In Table 4 we compared the performances of PhyloCys with Sephiroth [16] on those datasets. PhyloCys performances on OXCYS15 are comparable (+2%) to the ones obtained on IND-SPXnoFrag (see Table 3) but are 9–13% lower than the ones obtained on PDBCYS, even when ignoring the 21 empty phylogenetic trees (second row of OXCYS15 performances in Table 4). Sephiroth also experiences a drop in performance, with the average Qp 4% less than in PDBCYS. The OXCYSnr dataset is even more difficult to predict: the number of empty phylogenetic trees is 29 and PhyloCys performances are similar to the ones obtained on IND-SPX without the removal of the fragments. Sephiroth also experiences a 25% decrease in its average Qp score. Ignoring the proteins with empty phylogenetic trees or empty alignments provides respective improvements of 8% and 5% for both methods.

**Table 4:** PhyloCys performances on OXCYSnr and OXCYS15 datasets.

Dataset	Alignments		Number of Bonds								
		Empty MSAs/trees	2		3		4		5		Average
Num. Iter.	E-value		Rb	Qp	Rb	Qp	Rb	Qp	Rb	Qp	Qp
Random			33	33	20	7	14	1	11	0.1	15
PhyloCys	OXCYS15	21	73	73	51	42	42	20	39	15	48
PhyloCys	OXCYS15	0*	74	74	55	45	44	21	40	15	50
Sephiroth	OXCYS15	19	77	77	59	50	47	28	47	20	54
Sephiroth	OXCYS15	0*	77	77	61	52	48	29	48	21	55
PhyloCys	OXCYSnr	29	65	65	36	23	31	10	32	8	36
PhyloCys	OXCYSnr	0*	67	67	41	26	34	11	34	9	39
Sephiroth	OXCYSnr	26	72	72	41	29	37	19	36	14	43
Sephiroth	OXCYSnr	0*	73	73	44	90	37	20	37	15	45

PhyloCys performances on OXCYSnr and OXCYS15 datasets. All the performances have been obtained with hhBlits alignments using 3 iterations and E-value of 10^−5^. The symbol * indicates that those performances have been calculated without taking into account the empty MSAs.

The likely reason for this drop in the performance is apparent from the distributions of the number of homologs retrieved for the datasets (Fig 5). The median of the MSAs sizes for OXCYS15 is similar to PDBCYS and IND-SPXnoFrag, but higher than IND-SPX, while OXCYSnr has the lowest median MSA size (except for the fragments) with a slightly smaller variance. Both OXCYS15 and OXCYSnr contain relatively new PDB structures, which might be the cause for the observed scarcity of homologous sequences. Moreover, PhyloCys is more strongly affected than Sephiroth by the reduced MSA size because it is restricted to homologs having an even number of cysteines in the oxidized positions, thus further reducing the total number of available sequences. In particular, for both OXCYSnr and OXCYS15, the 20% of the collected homologs are discarded due to this parity issue.

### Relaxing the tandem mutation model

In the Methods section we described the algorithm for the conversion of the tandem mutations patterns evinced from the phylogenetic trees into edge weights for the disulfide connectivity graph. This procedure relates to different cases that can occur in the history of cysteine mutations in the phylogenetic tree. We described the most straightforward approach, in which we increase the weights of corresponding disulfide bonds every time a (single or multiple) tandem mutation is observed, but it is not always the case.

When *parity breaking* mutations occur, where an odd number of cysteines mutate in a single evolutionary step, possible tandem mutations are confounded by the odd number of mutating cysteines. In these cases we tried to divide the weight increment among the bonds that are possible between the odd number of mutating cysteines while the weights between the mutating and the conserved cysteines are decreased. The idea was to to exploit this unclear situation to determine which cysteines are *not likely* to have disulfide bonds between them because they are not involved in the mutations happening in the current step, but the extremely slight increment of the prediction performances was not sufficient to justify this over-complication of the model.

### PhyloCys applied to Machine Learning methods

The postulation that the information provided by evolution-based unsupervised predictors is to a certain extent orthogonal to the model learned by supervised Machine Learning (ML) methods [15] was previously confirmed by a ∼10% improvement of a Support Vector Regression method when coupled with MIp and ICOV predictions [9]. To investigate the usefulness of PhyloCys when applied to supervised prediction methods, we faithfully reproduced the same ML-based disulfide connectivity predictor adopted in [9–11] (see Methods) and we evaluated the contribution that our method can provide when its predictions are added to the feature vectors used by ML tools. We refer to this implementation as “skSVR”, with the performance comparison in Table 5 obtained from the same 20-fold cross-validation procedure performed for SVR+MIp+ICOV [9]. A “+” symbol indicates where the predictions obtained by an unsupervised method such as PhyloCys are added as an additional feature to skSVR. The skSVR+PhyloCys approach performs 24% better than the SVR presented in [9] alone and 14% better than SVR+MIp+ICOV. MIp+ICOV provide a +9% improvement with respect to their SVR alone and the relative enhancement provided by PhyloCys to skSVR is +12%, even if our skSVR implementation starts from performances that are already 11% better than in their SVR implementation. PhyloCys performs 1.5% less than Sephiroth [16] in terms of average Qp score, but provides a 18% improvement for the 4 bonds and a 11% improvement for the 5 bonds.

Finally, we performed the same 20-fold cross-validation on an implementation where both Sephiroth and PhyloCys predictions were added to the skSVR feature vectors. While our machine learning approach outperforms the older state of the art methods, it only marginally improves the skSVR+Sephiroth approach (+2.5% of overall Qp).

### Verifying the assumptions of the model: reconstruction of ancestral sequences

The basic assumptions on which the PhyloCys model of cysteine evolution is based are analogous to the ones postulate by most of bioinformatics tools that connect the evolution of sequence to that of structure. The PhyloCys model assumes in particular that (1) the disulfide connectivity is conserved within a protein family and (2) that once a cysteine is mutated, the probability of restoring the same cysteine with another mutation is negligible and thus not considered relevant by our model. In other words, cysteines can only be lost during the limited amount of evolutionary time we sample with our simplified method, and when traversing the phylogenetic tree from the leaves to the root the number of cysteines can never decrease.

We tried to assess the biological reliability of the assumption (2) qualitatively on example proteins by comparing the cysteine presence/absence patterns hypothesized by PhyloCys with the same positions predicted by FastML, a specifically designed ancestral sequence reconstruction tool [29]. We downloaded the FastML program and we used it to reconstruct the sequences corresponding to the inner nodes in the same trees used by PhyloCys. We were able to perform this comparison only for 35 proteins with less than 126 collected homologs each due to the exponential computational cost of the reconstruction procedure [29]. Even with this limitation we can compare the presence of absence of cysteines in ancestral sequences hypothesized by PhyloCys with the one predicted by FastML for 1609 inner nodes (on average of 45.97 per protein).

In 32 of the 35 protein families (91.4%) reconstructed by FastML oxidized cysteines are only lost during evolution. The 3 discordant families each have one single edge that does not respect assumption (2), which means that, in total, 3285 (99.9%) of the 3288 *parent-child* edges in these trees are consistent with our assumption.

On the other hand, FastML and PhyloCys do not assign exactly the same binary names to all the nodes in the examined trees: among the 35 reconstructed proteins, FastML and PhyloCys assign exactly the same label to 85% of the internal nodes (1370 out of 1609). PhyloCys assigns labels in order to maximize the parsimony with which it distributes mutations; it basically minimizes the Hamming distance between each parent and its two children. Therefore, the labels of the internal nodes can differ from FastML’s maximum likelihood based approach, even if the simplified model of evolution and assumption (2) are 99.9% respected within the boundaries of both models.

## Discussion

PhyloCys, a novel method for the unsupervised prediction of disulfide bond connectivity patterns, is rooted on the tandem mutation assumption used in other methods [9, 15], but instead of using patterns of co-variation between aligned homologs, Phylocys puts the homologous sequences in an evolutionary context: the ancestral sequences from a phylogenetic tree are used to to infer the disulfide bonding pattern. In terms of phylogeny, the homologous sequences represent the leaves of an evolutionary tree, which are connected by inner nodes that represent ancestral sequences. Phylocys assesses tandem cysteine mutations that occur *vertically* in the tree (between a node and its ancestors, at different depths in the tree), whereas other methods analyze a *flattened* version of this information, comparing only the *horizontal* variation among retrieved homologs (the leaves of the tree). PhyloCys then also takes into account the evolutionary distances between the sequences as perceived by the ClustalW Neighbor Joining algorithm. These distances are derived from whole sequences and the relationship between them, and not limited to positions containing oxidized cysteines.

In our previous Sephiroth work [16] we addressed this problem by clustering similar sequences and then inferring the disulfide bonding from them.

Both Sephiroth and Phylocys use distances between full sequences: the label of each internal node, and so the prediction procedure, is potentially influenced by all the other sequences; the tree is constructed by ClustalW from an evolutionary distance matrix containing all MSA entries. The PhyloCys performance is not better than Sephiroth, however, which we attribute to the removal of all homologous sequences with an odd number of cysteines: these sequences introduce noise in the inference performed by PhyloCys, whereas they do not affect Sephiroth. The effect is particularly noticeable for target sequences with very few homologs, where it causes a drop in the PhyloCys prediction reliability.

The new datasets we make available for training and testing should help stimulate new approaches in the cysteine bonding prediction field. We are however especially hopeful that other methods that predict protein characteristics from sequence, but which use homologous sequences horizontally, will benefit from the reasoning introduced in this paper and will start using the depth and whole sequence distances provided by evolutionary approaches.

## Supporting Information

S1 Fig Distributions of the sequence lengths in the datasets. Comparison of the distribution of the sequence lengths in SPX (green) and PDBCYS (red) datasets. SPX contains more very short sequences than PDBCYS
(EPS)

S2 Fig Distributions of the number of bonds in the datasets. Histogram showing the number of proteins with more than 2 disulfide bonds in SPX (green) and PDBCYS (red) datasets.
(EPS)

S3 Fig Distributions of the protein lengths in the datasets. Boxplot showing the comparison between the protein lengths distributions of PDBCYS and SPX dataset. SPX contains more shorter sequences.
(EPS)

S4 Fig Variation of PhyloCys performances in function of the number of homologs used on PDBCYS. The prediction performances for 2 to 5 bonds and their average change in function of the percentage of homologs from the PDBCYS dataset used to build the ClustalW phylogenetic trees.
(EPS)

S5 Fig Particular showing the distribution of the MSA sizes on the short sequences. Boxplot showing the distribution of the number of collected homologs for the 161 sequences in SPX with less than 40 residues. More than half of them have less than 100 homologs and the median is around 25 homologs for each protein.
(EPS)
